# Risk Factors for Hospitalization or Death Among Adults With Advanced HIV at Enrollment for Care in South Africa: A Secondary Analysis of the TB Fast Track Trial

**DOI:** 10.1093/ofid/ofac265

**Published:** 2022-06-09

**Authors:** Claire J Calderwood, Mpho Tlali, Aaron S Karat, Christopher J Hoffmann, Salome Charalambous, Suzanne Johnson, Alison D Grant, Katherine L Fielding

**Affiliations:** Clinical Research Department, Faculty of Infectious and Tropical Diseases, London School of Hygiene & Tropical Medicine, London, UK; Institute for Global Health, University College London, London, UK; The Aurum Institute, Johannesburg, South Africa; Clinical Research Department, Faculty of Infectious and Tropical Diseases, London School of Hygiene & Tropical Medicine, London, UK; Johns Hopkins University School of Medicine, Baltimore, Maryland, USA; Clinical Research Department, Faculty of Infectious and Tropical Diseases, London School of Hygiene & Tropical Medicine, London, UK; School of Public Health, Faculty of Health Sciences, University of the Witwatersrand, Johannesburg, South Africa; Foundation for Professional Development, Pretoria, South Africa; Clinical Research Department, Faculty of Infectious and Tropical Diseases, London School of Hygiene & Tropical Medicine, London, UK; School of Public Health, Faculty of Health Sciences, University of the Witwatersrand, Johannesburg, South Africa; Africa Health Research Institute, School of Laboratory Medicine and Medical Sciences, College of Health Sciences, University of KwaZulu-Natal, Durban, South Africa; Clinical Research Department, Faculty of Infectious and Tropical Diseases, London School of Hygiene & Tropical Medicine, London, UK; Department of Infectious Disease Epidemiology, Faculty of Epidemiology and Population Health, London School of Hygiene & Tropical Medicine, London, UK

**Keywords:** HIV, opportunistic infections, tuberculosis, anemia

## Abstract

**Background:**

Individuals with advanced HIV experience high mortality, especially before and during the first months of antiretroviral therapy (ART). We aimed to identify factors, measurable in routine, primary health clinic–based services, associated with the greatest risk of poor outcome.

**Methods:**

We included all individuals enrolled in the standard-of-care arm of a cluster-randomized trial (TB Fast Track); adults attending participating health clinics with CD4 ≤150 cells/µL and no recent ART were eligible. Associations between baseline exposures and a composite outcome (hospitalization/death) over 6 months were estimated using multivariable Cox regression.

**Results:**

Among 1515 individuals (12 clinics), 56% were female, the median age was 36 years, and the median CD4 count was 70 cells/μL. Within 6 months, 89% started ART. The overall rate of hospitalization/death was 32.5 per 100 person-years (218 outcomes/671 person-years). Lower baseline CD4 count (adjusted hazard ratio [aHR], 2.27 for <50 vs 100–150 cells/µL; 95% CI, 1.57–3.27), lower body mass index (aHR, 2.13 for BMI <17 vs ≥25 kg/m^2^; 95% CI, 1.31–3.45), presence of tuberculosis-related symptoms (aHR, 1.87 for 3–4 symptoms vs none; 95% CI, 1.20–2.93), detectable urine lipoarabinomannan (aHR, 1.97 for 1+ positivity vs negative; 95% CI, 1.37–2.83), and anemia (aHR, 4.42 for severe anemia [hemoglobin <8 g/dL] vs none; 95% CI, CI 2.38–8.21) were strong independent risk factors for hospitalization/death.

**Conclusions:**

Simple measures that can be routinely assessed in primary health care in resource-limited settings identify individuals with advanced HIV at high risk of poor outcomes; these may guide targeted interventions to improve outcomes.

Despite the expansion of access to antiretroviral therapy (ART), many people present to HIV services with advanced disease, particularly in Southern Africa [1]. These individuals experience the greatest burden of morbidity and mortality, particularly due to tuberculosis (TB) and other opportunistic infections (OIs) [2, 3], resulting in high health care costs [4]. However, several trials of empiric TB treatment have failed to demonstrate improved survival. Understanding the pathways leading to poor outcomes may aid development of novel approaches to improve outcomes and facilitate targeted interventions for the highest-risk individuals.

Lower CD4 count and/or advanced World Health Organization (WHO) stage, TB, and other OIs are all commonly described risk factors for death among people with HIV [2, 5–7]; ART improves outcomes [8]. Other risk factors include malnutrition [7], anemia [9, 10], and sociodemographic factors such as male gender [11, 12], older age [13–15], and lower socioeconomic position [16]. These factors have mostly been described among ART-naïve individuals at ART initiation and at large clinics or hospitals. Few studies have included ART-experienced individuals who are re-engaging with care; however, this group represents an increasing proportion of people attending HIV services with advanced disease [5, 10]. While mortality is decreasing among people with severe immunosuppression at ART initiation, likely due to improved clinical care, the first months of ART remain a period of exceptionally high risk of poor outcomes [5, 17].

Understanding and addressing drivers of mortality among people with advanced HIV as early as possible after entering HIV care is of critical importance in improving overall outcomes among this group [18]. This analysis aimed to identify risk factors, measurable at entry to HIV care among adults with advanced HIV in routine primary health clinics (PHC) in South Africa, for increased rate of the composite outcome of death or hospitalization over the subsequent 6 months and to explore the underlying causal pathways by which these factors contribute to individual outcomes.

## METHODS

### Study Setting and Design

This analysis drew on data from the TB Fast Track trial; a pragmatic, 2-arm cluster-randomized trial in South Africa, employing a nurse-led risk stratification algorithm to guide empiric TB treatment [19]. To reflect the experience of individuals in routine care, data from the standard-of-care arm of the trial were used [19, 20]. Briefly, adults (aged ≥18 years) with HIV who attended 12 participating clinics in 3 provinces of South Africa (Limpopo, North West, and 2 districts in Gauteng) with available CD4 results were invited to participate. Included individuals were willing to start ART and had a CD4 count of ≤150 cells/µL. Exclusion criteria were recent TB treatment (past 3 months) or ART (past 6 months), contraindication to first-line ART, or being too unwell at the first visit to be managed in primary care (defined in the Supplementary Data). Of 1579 individuals screened (December 2012–December 2015), 1515 individuals (96%) were enrolled [19].

### Patient Consent

TB Fast Track received ethical approval from the University of the Witwatersrand, South Africa, and London School of Hygiene & Tropical Medicine, United Kingdom, and informed written consent was obtained from all participants.

### Outcome and Exposures of Interest

A composite outcome of hospitalization or death was used. Individuals were censored at the first of either event, the last date known to be alive, or the end of follow-up (6 months). Robust measures were in place to trace individuals regardless of retention in HIV care. Participants were asked to contact the study team in case of hospitalization. Participant or nominated relative (for those who died) interview at 6 months included questions about dates of hospitalization events and death. Data were additionally abstracted from clinical case note reviews at 2 and 6 months and hospital record reviews. Data were linked to vital registration systems for participants with unknown outcomes at 6 months [19].

Exposures of interest were sociodemographic characteristics (age, gender, socioeconomic position), CD4 count, body mass index (BMI), current TB, cryptococcal antigenemia (CrAg), and anemia. BMI and anemia were analyzed as categorical variables, per WHO definitions, to facilitate clinical interpretation of associations (Supplementary Table 1.1 and 1.2) [21, 22]. We considered several measures associated with TB disease: TB-related symptoms (as defined by the WHO symptom screen: fever, weight loss, night sweats, and cough [23]), urine lipoarabinomannan (Determine TB LAM, Alere Inc, Waltham, MA, USA [LAM]; post–January 2014 reference standard), and sputum-based TB tests (Xpert MTB/RIF or acid-fast bacilli smear). LAM was measured retrospectively after study completion for all participants with semiquantitative grading against the post–January 2014 reference standard (unlike the primary trial analysis) [24]; 3+ and 4+ intensity bands were grouped due to data sparsity. Sputum-based TB tests were only performed if required as part of routine care. Hemoglobin was recorded where performed as part of routine care. CD4 count and age were modeled as categorical variables by dividing into thirds or quartiles, respectively, after exploration of the functional form of this association using fractional polynomials. All participants with an adequate dried blood spot sample had blood retrospectively assayed for CrAg. Routine testing was additionally performed as per national guidelines, where a recommendation for CrAg among people with HIV and CD4 <100 cells/µL was introduced while the trial was ongoing. Dates of TB treatment, ART, isoniazid preventative therapy (IPT), and co-trimoxazole preventative therapy (CPT) initiation were recorded. National guidelines advised ART initiation 2–8 weeks after starting TB treatment; same-day ART (for people without TB) was not included in national guidelines [25].

### Statistical Analysis

**Figure 1.**
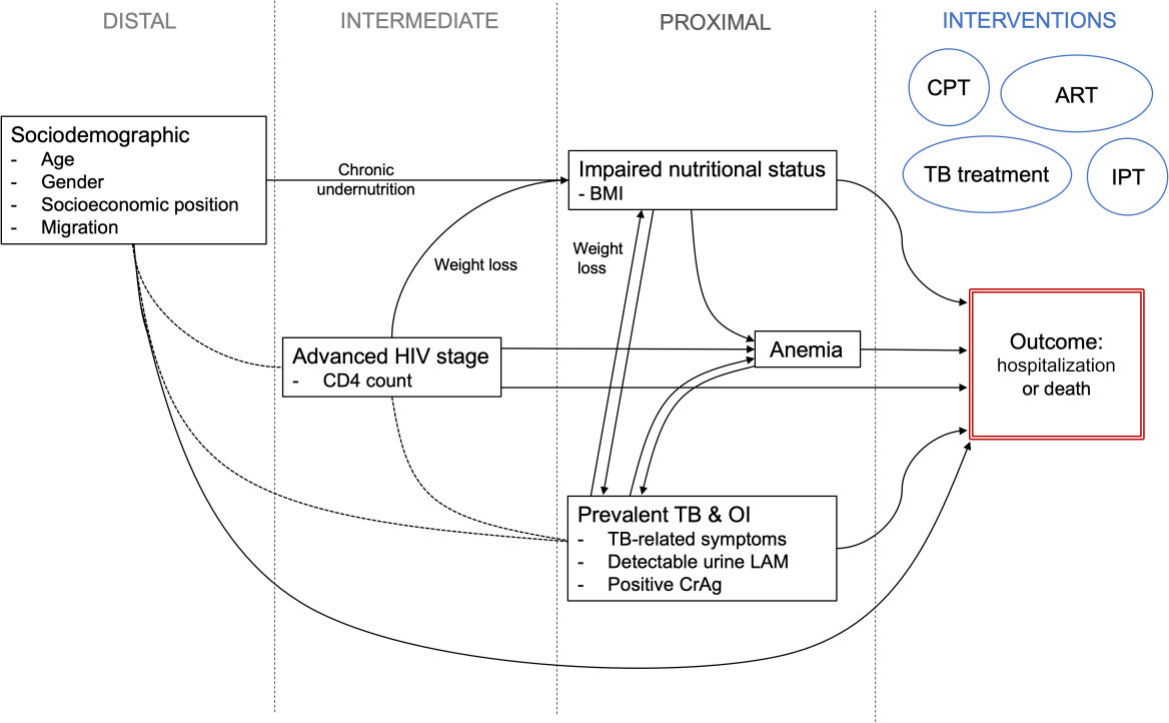
Conceptual framework outlining proposed associations and causal pathways between risk factors of interest and hospitalization/death. Risk factors of interest were considered to be distal, intermediate, or proximal in their association with the outcome of hospitalization or death, and these relationships were used to develop a model estimating the total effect of each factor (adjusting for distal and intermediate factors; Model A) and a model estimating the direct effect of each factor (adjusting for all other factors; Model B). Solid arrows indicate proposed causal pathways, and dotted lines indicate associations. Age, sex, and socioeconomic position (measured here by assets owned, type of housing, and employment) influence nutritional status (reflected in BMI) and HIV stage at entry to care (CD4 count) and thus were considered distal factors. Advanced HIV (assessed by CD4 count) causes acute weight loss (resulting in lower BMI), anemia, and higher risk of active TB (indicated by TB-related symptoms, positive LAM, or other TB tests [Xpert MTB/RIF, sputum acid-fast bacilli smear, and chest radiograph]), with greater severity at lower CD4 counts. TB-related symptoms may also reflect the presence of opportunistic infections (OIs), which cause symptoms that overlap with TB. Active TB disease and OIs also cause weight loss and anemia, while malnutrition increases the risk of TB disease and anemia [26]. In our population, lower BMI may therefore reflect long-term undernutrition or weight loss attributable to disease. Lower BMI, anemia, and active TB disease or OI at entry to care are proximate factors, proposed to have causal relationships with the outcome of hospitalization or death over the subsequent 6 months. For clarity of presentation, associations between interventions (indicated by circles) with other factors of interest and the outcome are not presented. Each intervention has been demonstrated to reduce mortality and complications in individuals with advanced HIV from randomized controlled trials [9–9]. Abbreviations: ART, antiretroviral therapy; BMI, body mass index; CPT, co-trimoxazole preventative therapy; CrAg, cryptococcal antigen; IPT, isoniazid preventative therapy; LAM, urine lipoarabinomannan; OI, opportunistic infection; TB, tuberculosis.

Analyses were conducted using Stata, version 15 (StataCorp, College Station, TX, USA). Cox proportional hazards regression was used to estimate associations between exposures and the outcome. Multivariable models were developed using a forward approach, with age and gender included a priori. Factors were grouped into distal, intermediate, and proximal, with relationships as described in Figure 1. In view of complex relationships between exposures of interest, 2 models were developed. Model A estimated the total effect of each factor of interest, adjusting for distal and intermediate factors. Model B included all identified risk factors to describe direct effects [30]. The Stata code used for each model is provided in the Supplementary Data.

We assessed the proportional hazards assumption and collinearity in both multivariable models. Clinic-level clustering was accommodated using a fixed effect for district (4-level variable). Likelihood ratio tests (LRTs) were conducted for overall association, linear trend, and departures from linearity.

A complete case record approach was employed. There were considerable missing data for hemoglobin, because at 3 clinics ≤25% of participants underwent testing. Analyses of anemia were therefore restricted to 9 clinics with routine hemoglobin testing. A socioeconomic position score was generated using principal component analysis; the score was grouped into quintiles (Supplementary Table 1.3). Population-attributable fractions (PAFs) were calculated using estimates from multivariable models [31].

The diagnostic tests available in TB Fast Track may not be available in routine clinical care. As a sensitivity analysis, we explored the impact of including these tests in our model, repeating the analysis considering only clinical factors.

## RESULTS

### Study Population

A total of 1515 participants across 12 clinics were included, with few missing data aside from hemoglobin (29% missing overall, 7% when restricted to 9 clinics) (Table 1). Fifty-six percent (849/1515) of participants were female, the median age (interquartile range [IQR]) was 36 (18–83) years, and the median CD4 count (IQR) was 70 (35–113) cells/µL.

**Table 1.** Distribution of Sociodemographic Factors at Baseline and Rates and Univariable Hazard Ratios for Time to Hospitalization/Death (n = 1515)

		No. (%)	Events/PY	Rate/100PY	HR^a^	95% CI	*P*
	Overall	1515	218/671	32.5			
Sex (n = 1515)	Female	849 (56)	113/380	29.8	Ref		.1
	Male	666 (44)	105/292	36.0	1.22	(0.93–1.60)	
Age, y (n = 1515)	18–29	290 (19)	33/130	25.29			.2
	30–44	910 (60)	142/399	35.56	1.40	(0.96–2.05)	
	≥45	315 (21)	43/141	30.42	1.21	(0.77–1.90)	
Country of origin (n = 1514)	South Africa	1373 (91)	201/610	32.9	Ref		.5
	Other SSA	141 (9)	17/61	28.1	0.85	(0.51–1.41)	
SEP quintile (n = 1489)	1 (lowest)	298 (20)	44/131	33.6	Ref		.8
	2	298 (20)	37/133	27.9	0.84	(0.54–1.30)	
	3	298 (20)	44/132	33.4	1.00	(0.66–1.53)	
	4	298 (20)	48/132	36.3	1.11	(0.73–1.67)	
	5 (highest)	297 (20)	40/132	30.3	0.94	(0.60–1.47)	
CD4 count, cells/µL (n = 1515)	<50	522 (34)	113/217	52.1	2.63	(1.86–3.72)	<.001^b^
	50–99	496 (33)	60/221	27.2	1.40	(0.95–2.06)	
	>100	497 (33)	45/233	19.3	Ref		
BMI, kg/m^2^ (n = 1512)	<17	141 (9)	36/58	62.4	2.89	(1.81–4.59)	<.001^b^
	17–18.4	130 (9)	22/56	39.5	1.84	(1.08–3.13)	
	18.5–24.9	872 (58)	122/386	31.6	1.47	(1.01–2.12)	
	≥25	369 (24)	37/171	21.7	Ref		
TB-related symptoms (n = 1511)	None	518 (34)	50/242	20.7	Ref		<.001^b^
	1	491 (33)	61/220	27.8	1.35	(0.92–1.96)	
	2	302 (20)	66/124	53.1	2.55	(1.76–3.71)	
	3+	200 (13)	41/84	49.0	2.42	(1.59–3.70)	
TB tests performed^c^ (n = 1515)	None	1265 (84)	184/561	32.8	Ref		.1
	Negative	195 (13)	21/89	23.7	0.71	(0.45–1.13)	
	Positive	31 (2)	7/13	55.9	1.68	(0.79–3.59)	
	Unknown	24 (2)	6/9	65.0	1.85	(0.82–4.19)	
Previous TB treatment (n = 1515)	No	1379 (91)	196/611	32.1	Ref		.6
	Yes	136 (9)	22/60	36.7	1.15	(0.74–1.78)	
Previous TB test (≤6 mo) (n = 1515)	No	873 (58)	118/390	30.2	Ref		.2
	Yes	642 (42)	100/281	35.6	1.18	(0.90–1.55)	
LAM^d^ (n = 1463)	Negative	1241 (85)	146/565	25.9	Ref		<.001^b^
	1+	162 (11)	38/65	58.6	2.22	(1.55–3.17)	
	2+	31 (2)	11/11	98.2	3.71	(2.01–6.87)	
	3–4+	29 (2)	13/9	150.6	5.56	(3.14–9.84)	
Serum CrAg^d^ (n = 1410)	Negative	1399 (99)	207/618	29.5	Ref		.6
	Positive	11 (1)	1/5	20.2	0.62	(0.09–4.43)	
Current CPT (n = 1514)	No	798 (53)	106/355	29.9	Ref		.2
	Yes	716 (47)	112/316	35.5	1.19	(0.89–1.57)	
Current IPT (n = 1514)	No	1356 (90)	197/598	33.0	Ref		.5
	Yes	158 (10)	21/73	28.9	0.86	(0.54–1.37)	
Restricted data set (9 clinics)							
Anemia^e^ (n = 1021/1099)	Severe	94 (9)	28/36	77.1	4.86	(2.73–8.66)	<.001^b^
	Moderate	380 (37)	64/166	38.6	2.52	(1.52–4.17)	
	Mild	260 (26)	37/116	31.9	2.07	(1.20–3.58)	
	None	287 (28)	20/134	14.9	Ref		

Anemia was categorized according to WHO definitions (severe anemia = Hb <80 g/dL; moderate anemia = Hb ≥80 and <110 g/dL; mild anemia = Hb ≥110 and <130 [if male] or <120 [if female]; none = Hb ≥130 [if male] or ≥120 [if female]).

Abbreviations: BMI, body mass index; CPT, co-trimoxazole preventative therapy; CrAg, cryptococcal antigen; Hb, hemoglobin; HR, unadjusted hazard ratio; IPT, isoniazid preventative therapy; LAM, urine lipoarabinomannan; LRT, likelihood ratio test; n, number of individuals with nonmissing data for this variable; ref, reference category; *P*, *P* value from likelihood ratio test for association; PY, person-years at risk; SEP, socioeconomic position; SSA, Sub-Saharan Africa; TB, tuberculosis; WHO, World Health Organization.

a HR from Cox regression model adjusted for clustering using fixed effect for district.

b *P* < .001 from LRT for linear association; no evidence for departure from linearity (*P* > .3) apart from for number of TB-related symptoms where *P* = .09.

c These were either sputum acid-fast bacilli smear or Xpert MTB/RIF within 0–14 days after study enrollment.

d No individuals were LAM positive at the 5+ intensity band; 52 and 105 individuals did not have an adequate sample for LAM and CrAg testing, respectively.

e Anemia analyses use a restricted data set as described in text. Overall, 1080/1515 individuals had hemoglobin measured.

**Figure 2.**
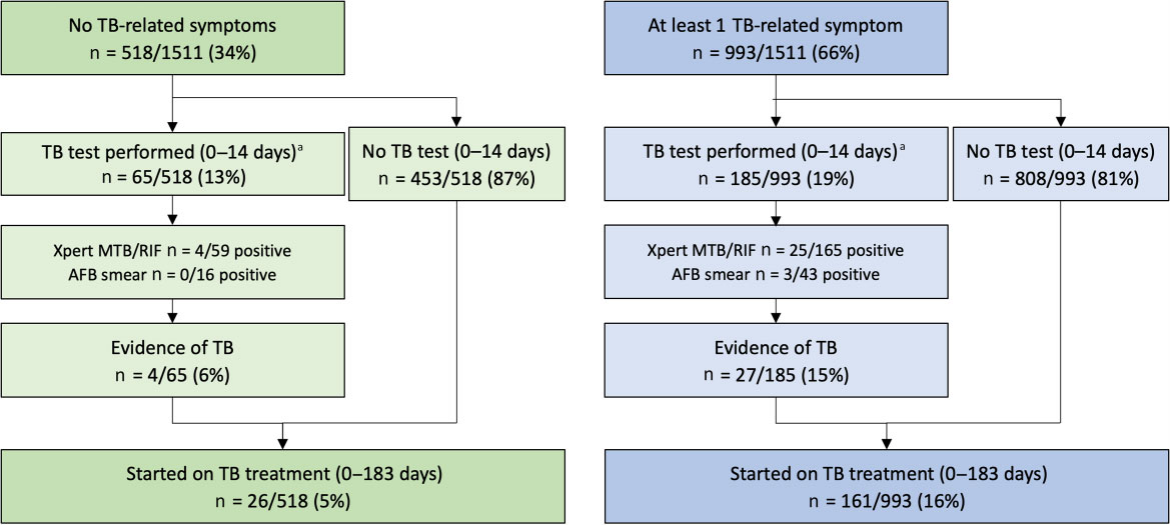
Overview of TB-related symptoms, TB tests performed, and results among participants (N = 1515). Data on the presence of TB-related symptoms were not available for 4 participants. This assessment was separate from the routine clinical assessment performed by clinic staff. Use of symptom screening for TB, TB diagnostic tests, and initiation of TB treatment were all as per usual clinic practice. ^a^Of all TB tests performed 0–14 days from enrollment, 117/250 (47%) were performed on the day of enrollment (93 among people reporting at least 1 TB-related symptom). Sixteen of 117 (14%) tests performed on the day of enrollment showed evidence of TB (Xpert MTB/RIF: n = 14/103 positive), AFB smear (n = 3/28 positive). Abbreviations: AFB, microscopy for acid-fast bacilli; CXR, chest x-ray; TB, tuberculosis.

At enrollment, 65% (992/1511) of participants reported at least 1 TB-related symptom: 59% had weight loss, 33% cough, 20% night sweats, 16% fever. TB tests were performed in 250 participants at enrollment or in the following 2 weeks; 31/250 (12%) had evidence of TB (Figure 2).

Urine LAM (performed after study completion) was detectable in 15% of participants (222/1463 with an adequate sample), with 4% (60/1463) positive at the 2+ level. Less than 1% of participants were CrAg positive (11/1410 with an adequate sample). Forty-seven percent (716/1514) were taking CPT and 10% (158/1514) IPT at enrollment, with initiation at a median (IQR) interval of 2 (–10 to 0) days or 1 (–10 to 0) day before enrollment, respectively.

Over 6 months, 89% of participants started ART and 12% of participants started TB treatment. The median interval from enrollment to ART (IQR) was 11 (5–21) days, and the median interval (IQR) to starting TB treatment was 6 (1–17.5) days. The interval between enrollment and ART was longer among those who also started TB treatment compared with those who did not (median [IQR], 29 [15–55] and 9 [4–19] days, respectively). Ninety-seven percent (1258/1297) of those who did not experience the outcome completed at least 5 months of follow-up. The overall mortality rate was 24.4 per 100 person-years (95% CI, 17.3–34.6).

### Rate of Hospitalization or Death

Two hundred eighteen individuals were hospitalized or died over 671 person-years of follow-up (comprising 60 deaths and 158 hospitalizations; 91 individuals censored at hospitalization died within 6 months of enrollment). The overall rate of hospitalization/death was 32.5 per 100 person-years; 66.0 per 100 person-years before and 22.5 per 100 person-years after initiation of ART (95% CI, 46.7–93.3 and 16.1–31.4, respectively).

### Risk Factors for Hospitalization or Death

Univariable analyses showed little evidence for associations between sociodemographic measures and rate of hospitalization/death (Table 1). Strong evidence for univariable associations between each of CD4 count, BMI, TB-related symptoms, detectable urine LAM, and anemia was observed (Table 1; Supplementary Figure 1).

In multivariable analyses, lower CD4 count, lower BMI, presence of TB-related symptoms, detectable LAM, and more severe anemia were strong risk factors for shorter time to hospitalization/death (Table 2). After adjustment for sex, age, and district (model A), lower CD4 count was strongly associated with increased rate of hospitalization/death, with 2.7 times the hazard in those with CD4 <50 cells/µL compared with those with CD4 100–150 cells/µL (95% CI, 1.86–3.82; aHR, 1.65; 95% CI, 1.38–1.97; per 50 cell/µL category decrease).

**Table 2.** Adjusted Hazard Ratios for Hospitalization/Death From Multivariable Analysis (n = 1456)

	Model A^a^			Model B^a,b^				
	aHR	95% CI	*P*	aHR	95% CI	*P*	PAF, %^c^	95% CI, %
CD4 count, cells/µL								
<50	2.66	(1.86–3.82)	<.001^d^	2.27	(1.57–3.27)	<.001^d^	38	(20–53)
50–99	1.51	(1.02–2.25)		1.53	(1.02–2.28)			
100–150	Ref	…		Ref	…			
BMI, kg/m^2^								
<17	2.64	(1.64–4.24)	.001^d^	2.13	(1.31–3.45)	.02^e^	26	(1–45)
17–18.4	1.79	(1.02–3.12)		1.61	(0.92–2.82)			
18.5–24.9	1.40	(0.96–2.05)		1.31	(0.90–1.92)			
≥25	Ref	…		Ref	…			
LAM								
Negative	Ref	…	<.001^d^	Ref		<.001^d^	17	(9–24)
1+	2.05	(1.43–2.95)		1.97	(1.37–2.83)			
2+	3.68	(1.97–6.85)		3.09	(1.65–5.78)			
3–4+	4.20	(2.33–7.55)		3.46	(1.92–6.26)			
TB-related symptoms								
0	Ref	…	<.001^d^	Ref	…	<.001^d^	29	(8–45)
1	1.23	(0.83–1.80)		1.19	(0.81–1.76)			
2	2.32	(1.58–3.42)		2.08	(1.41–3.07)			
≥3	2.09	(1.34–3.25)		1.87	(1.20–2.93)			
Restricted data set (9 clinics, n = 982)								
Anemia								
Severe				4.42	(2.38–8.22)	<.001^d^	48	(34–80)
Moderate				2.09	(1.24–3.55)			
Mild				1.79	(1.03–3.09)			
None				Ref				

Anemia was categorized according to WHO definitions (severe anemia = Hb <80 g/dL; moderate anemia = Hb ≥80 and <110 g/dL; mild anemia = Hb ≥110 and <130 [if male] or <120 [if female]; none = Hb ≥130 [if male] or ≥120 [if female]).

Abbreviations: aHR, adjusted hazard ratio; BMI, body mass index; Hb, hemoglobin; LAM, urine lipoarabinomannan; LRT, likelihood ratio test; *P*, *P* value from LRT for association; PAF, population-attributable fraction; Ref, reference category; TB, tuberculosis; WHO, World Health Organization.

a Model A: Five different models assessing the association between each variable shown and the outcome, in each case adjusted for sex, age, CD4 count, and district and restricted to observations also included in model B. Model B: adjusted for all other variables in the table, age, and sex, using a fixed effect for district. Linear terms were used for BMI, number of TB-related symptoms, and LAM (with an indicator variable) in anemia analyses.

b In model B, for male (vs female): aHR, 0.92 (95% CI, 0.69–1.23; *P* = .6). For age 30–44 years and ≥45 years (vs 18–29 years): aHR, 1.39 (95% CI, 0.93–2.09) and 1.43 (95% CI, 0.89–2.32), respectively (*P* = .2).

c PAF for observed situation compared with a hypothetical scenario where all participants were in the reference category, assuming a causal association between each risk factor and the outcome.

d *P* < .001 for linear trend and *P* > .1 for departure from linearity from LRT apart from number of TB-related symptoms, where *P* = .09 for departure from linearity.

e *P* = .002 for linear trend and .98 for departure from linearity by LRT.

Lower BMI was strongly associated with shorter time to outcome after adjustment for CD4 count, sex, age, and district (model A): Those who were moderately to severely underweight (BMI <17 kg/m^2^) experienced over twice the rate of hospitalization/death compared with those in the highest BMI category (BMI ≥25 kg/m^2^; aHR, 2.66; 95% CI, 1.86–3.82).

Individuals with TB-related symptoms had a higher rate of hospitalization/death compared with those without, with greater hazard for each additional symptom reported. Individuals with 3 or more symptoms experienced over double the rate of these outcomes compared with those with none (aHR, 2.09; 95% CI, 1.34–3.25).

LAM positivity at 1+ intensity band was associated with a doubling in hazard compared with a negative result (HR, 2.05; 95% CI, 1.43–2.95), with a graded association across increasing levels of positivity (aHR, 1.72 per category increase from 1+ to 3–4+; 95% CI, 1.46–2.02).

In model B, all potential risk factors identified were included, irrespective of proposed mediating pathways. This demonstrated strong evidence for independent associations for each of CD4, BMI, LAM, and number of TB-related symptoms with the outcome. The effect size associated with the lowest CD4 counts (<50 cells/µL) was slightly smaller than observed in model A, suggesting mediation of the effect of CD4 count through lower BMI and/or markers of TB disease and OI. Similarly, when adjusted for TB-related symptoms, TB tests, and LAM, the magnitude of the hazard associated with being severely underweight (BMI <17 kg/m^2^) was smaller. When only clinical factors were considered, findings were similar (Supplementary Table 2.1).

In the data set restricted to 9 clinics for which hemoglobin data were available, anemia was strongly associated with increased rate of hospitalization/death after adjustment for sex, age, CD4 count, BMI, LAM, TB-related symptoms, and district. Those with severe anemia had over 4 times the hazard of hospitalization/death compared with none, with an aHR of 1.55 (95% CI, 1.27–1.89) per category increase in severity.

There was evidence for violation of the proportional hazards assumption in the association of baseline CD4 count with the outcome, with the increased hazard associated with lower CD4 count at study enrollment lessening over time (*P* = .03) (Table 3). For other risk factors considered, the proportional hazards assumption was met.

**Table 3.** Adjusted Hazard Ratios and Associated 95% CIs for Association Between Baseline CD4 Count and Time to Hospitalization/Death From Multivariable Analyses, Stratified by Period From Enrollment (n = 1456)

Duration of Follow-up	Overall (N = 207)	Month 1 (n = 90)	Month 2–3 (n = 71)	Month 4–6 (n = 46)
Baseline CD4 Count, cells/µL	aHR (95% CI)	aHR (95% CI)	aHR (95% CI)	aHR (95% CI)
<50	2.66 (1.86–3.82)	5.47 (2.69–11.2)	2.10 (1.19–3.71)	1.64 (0.85–3.17)
50–99	1.51 (1.02–2.25)	3.75 (1.79–7.87)	1.05 (0.57–2.09)	0.66 (0.29–1.50)
100–150	Ref	Ref	Ref	Ref

*P*_interaction_ = .02. Hazard ratios were adjusted for age, sex, and district presented (ie, model A above). In a model adjusted for age, sex, body mass index, number of tuberculosis symptoms, urine lipoarabinomannan, and district (ie, model B above), a similar association was observed (*P*_interaction_ = .02).

Abbreviations: aHR, adjusted hazard ratio; n = number of outcomes observed in the time period indicated.

### Population-Attributable Fractions

PAFs were calculated assuming independent causal associations with the outcome and adjusted for all other potential risk factors (Table 2). CD4 count <100 cells/µL and presence of anemia appeared to have the greatest population impact on rates of hospitalization/death (PAF 38% and 48%, respectively; 95% CI, 20%–53% and 34%–80%).

## DISCUSSION

Adults with advanced HIV in South Africa are at very high risk of hospitalization or death within 6 months of enrolling in care, particularly in the period before ART initiation. Within this population, clinical assessments and simple tests were strong risk factors for adverse outcomes, with independent associations between each of anemia, lower CD4 count, lower BMI, detectable urine LAM, presence of TB-related symptoms, and hospitalization/death. The greatest effect size was seen with positive LAM and anemia, but the greatest population impact could be attributed to lower CD4 count and anemia, reflecting the high prevalence of these factors among the study population.

Identification and management of people with advanced HIV remain critically important to improving individual outcomes. Despite rollout of earlier ART initiation, presentation with advanced disease remains common in South Africa [32]. Current recommendations for differentiated care for people with advanced HIV disease include prioritization for rapid initiation on ART. However, TB-related symptoms are common, often leading to a delay in ART [33]. Such delays may underlie the lack of benefit demonstrated by empiric TB treatment trials among people with advanced HIV, such as TB Fast Track [19]. Same-day ART initiation, despite TB-related symptoms, may improve outcomes by reducing the “pre-ART” period; however, evidence for specific approaches which facilitate this is currently lacking [34].

Rates of adverse outcomes among people with advanced HIV are not fully mitigated by ART initiation or explained by lower CD4 count. This, together with identification of BMI, anemia, LAM, and TB-related symptoms as risk factors for hospitalization/death, suggest that other strategies that directly address these risks may offer additional benefit. Such strategies may include rifamycin-containing TB-preventative therapy [35] and enhanced antimicrobial prophylaxis [36].

The pathways toward adverse outcomes among people with advanced HIV are, however, complex and interdependent. Such mediation is illustrated in our analysis by smaller direct effects (model B) as compared with total effects (model A) for all factors considered. This may suggest that a strategy targeting a single pathway may be insufficient and that further development and evaluation of multicomponent interventions, including both medical and nonmedical (eg, adherence counseling, financial support) elements, are required.

In this analysis, there was no association between IPT and CPT and hospitalization/death, despite clear evidence of reduced mortality with these strategies from randomized controlled trials [27]. The apparent lack of benefit may reflect low coverage, with fewer than half of participants taking CPT and only 10% IPT at enrollment, or insufficient duration of therapy, with most of those who were taking CPT/IPT starting around the time of enrollment, but the majority of adverse outcomes occurring early in follow-up. Finally, this finding may also reflect misclassification of exposure as data on CPT/IPT initiation after enrollment are not available.

In our study, simple tests (LAM, CD4, and hemoglobin) identified the people at highest risk of poor outcomes, with larger effect sizes and population-attributable fractions compared with clinical factors. LAM for TB diagnosis among people with HIV was not in routine use at the time of this study; however, 14% of outpatients with advanced HIV had detectable LAM, and +1 LAM positivity was associated with double the rate of hospitalization/death compared with a negative result. This further supports the systematic use of LAM in outpatient HIV care [37]. Measurement of CD4 is becoming less common in the era of test-and-treat; however, we suggest that it may continue to have a role in guiding differentiated care.

Despite commercially available cheap, battery-powered, or lateral flow point-of-care assays, tests for anemia or LAM are frequently not available in routine care. Improving access to such diagnostics may improve outcomes by promoting individualized care, enabling specific interventions to be tailored toward those most likely to benefit, or identifying people who require closer clinical monitoring or increased adherence or social support. Evaluation of diagnostics should be included in interventional studies for advanced HIV, particularly in the context of recent trials, which have not demonstrated benefit of interventions when applied to all “advanced HIV” [38, 39].

Many people with TB-related symptoms did not start TB treatment. This may reflect low specificity of symptom screening, but missed TB diagnoses due to limited availability and sensitivity of diagnostic tests are common, and better access to diagnostics (including routine use of LAM) may be important [40, 41]. Eighty-seven percent of people reporting TB-related symptoms had not had a sputum-based TB test 2 weeks later, perhaps suggesting lack of routine symptom-based TB screening, nondisclosure of symptoms to clinic staff, or positive symptom screens not being acted on, for example, due to difficulties accessing tests. It is possible that participants had TB tests and/or treatment initiation at a nonstudy health facility that were not captured. Both TB and other OIs cause TB-related symptoms. In a TB Fast Track substudy, 34 participants who died had minimally invasive autopsy: 47% had evidence of TB disease, 38% of those had not been on TB treatment, and multiple infections were common, mostly TB and bacterial infection [3].

The identified risk factors are generally consistent with those from other studies, though some findings differ [17]. Two systematic reviews concluded that men initiated on ART in Africa have higher mortality than women; no association was seen here [11, 12]. Univariable point estimates suggested increased hazard of hospitalization/death among men, though evidence was weak, and this was not seen after adjustment for more proximate factors, suggesting no direct effect. A number of studies included in the meta-analyses did not adjust for such confounding. Socioeconomic differences have been described as risk factors for HIV mortality [16]. Here, no trend was observed, perhaps reflecting a relatively homogenous, economically disadvantaged population. The data used in our analysis included data collected before ART initiation, which may also account for some of the differences compared with previous work.

The strengths of this analysis include the large sample size, few missing data, and complete follow-up, including for individuals not retained in care. Careful consideration of causal pathways facilitated estimation of total and direct effects of each risk factor and adjustment for confounding. TB Fast Track had few exclusion criteria; was based in PHCs; included individuals at receipt of CD4 results, not ART initiation; and, unlike most previous studies, was not restricted to ART-naïve individuals. This reflects real-world experience. We did, however, restrict to CD4 ≤150 cells/µL, rather than the standard definition of advanced HIV (200 cells/µL), and therefore cannot conclude that identified risk factors apply to less severe immunosuppression.

An important consideration when interpreting these findings is that individuals must not have received TB treatment in the previous 3 months to be enrolled, but 42% reported a TB test in the past 6 months. This was higher than expected and may be attributable to Xpert rollout during the study period. Aside from misclassification, recall, and social desirability bias, it must be assumed that these tests were negative. This exclusion is likely to have led to artificially low rates of TB disease; however, data from the autopsy substudy suggest similar TB prevalence among those who died to that found in other studies from the region [3, 42]. Individuals who did not attend to receive their CD4 result were not included in the study; it is likely that a significant number of this group did not start ART and therefore they may have been at higher risk of poor outcomes or may have had a different profile of risk factors.

As we considered only baseline risk factors, another limitation is potential time-dependent confounding; this may underlie the violation of proportional hazards observed in the case of CD4 count. CD4 counts may change rapidly, particularly when ART is initiated. As a result, an individual’s CD4 count at the time they experienced the outcome may be different from baseline.

In keeping with the pragmatic design of the trial, a limited number of variables were recorded, with possible resultant unmeasured confounding. In estimation of PAFs, we assumed a causal link between risk factors and the outcome; however, it is important to recognize in interpretation of these estimates that the effects of the factors described are mediated through other pathways such as infections and inflammatory changes, and they are not direct causes of the outcomes described. Further, residual or unmeasured confounding in these observational data is possible. Given the limited number of clusters, we did not attempt to evaluate clinic-level factors, which may also be important.

A composite outcome was selected to increase study power. Given very high mortality among hospitalized patients with advanced HIV, we proposed that hospitalization and death are both clinically important and that risk factors are likely to be similar for both [43]. Three-quarters of participants experiencing the outcome were censored at hospitalization: use of a composite outcome may have obscured factors associated with death but not hospitalization. However, 29% of hospitalized individuals died within 10 days of this event, suggesting that hospitalization is a relevant measure of severe morbidity.

## CONCLUSIONS

Individuals entering HIV care with advanced disease in South Africa have high morbidity and mortality. Here, clinical measures that may be routinely assessed in decentralized HIV services were identified as risk factors for the worst outcomes among this already high-risk group. As well as highlighting the ongoing need for earlier HIV diagnosis and ART to avoid advanced disease, this analysis suggests that simple clinical measures may enable differentiated care for individuals at the highest risk and therefore reduce the unacceptably high mortality in this group.

## Supplementary Material

ofac265_Supplementary_Data

## Supplementary Data

Supplementary materials are available at *Open Forum Infectious Diseases* online. Consisting of data provided by the authors to benefit the reader, the posted materials are not copyedited and are the sole responsibility of the authors, so questions or comments should be addressed to the corresponding author.
