# Supplementary material for: Risk Factors for Hospitalization or Death Among Adults With Advanced HIV at Enrollment for Care in South Africa: A Secondary Analysis of the TB Fast Track Trial
Source: Open Forum Infect Dis. 2022 Jun 9;9(7):ofac265. doi: 10.1093/ofid/ofac265 (PMC9290545; doi:10.1093/ofid/ofac265)
Supplement: ofac265_Supplementary_Data [file ofac265_supplementary_data.zip › tbft_rf_suppfigure_20220321_v5.docx]

**Supplementary material**

**Risk factors for hospitalisation or death among adults with advanced HIV at enrolment for care in South Africa: a secondary analysis of the TB Fast Track trial**

**Supplementary figure 1:** Kaplan-Meier curves illustrating hospitalisation/death events over 6 months of follow up, stratified by key risk factors for poor outcomes.

| **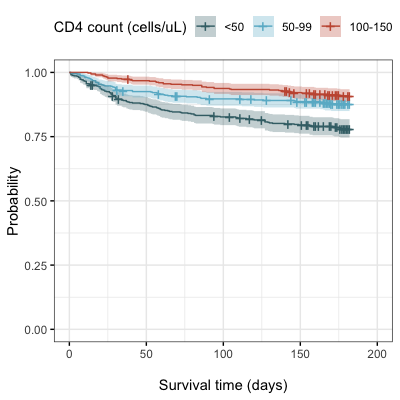** | **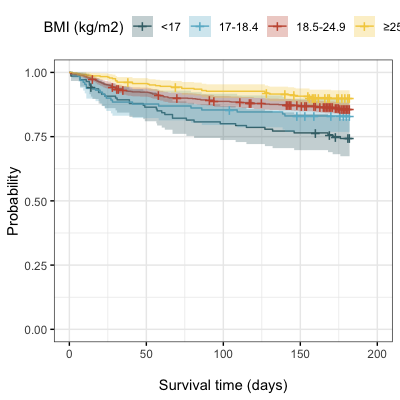** |
| --- | --- |
| **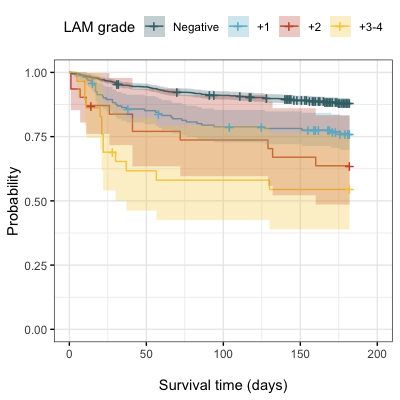** | **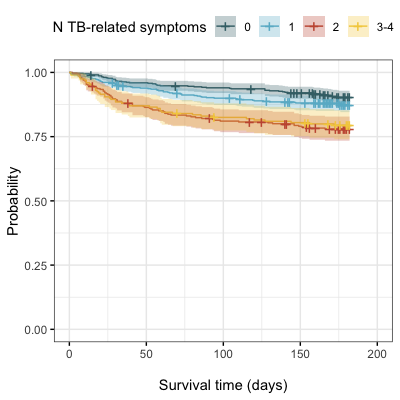** |
