# Supplementary material for: Risk Factors for Hospitalization or Death Among Adults With Advanced HIV at Enrollment for Care in South Africa: A Secondary Analysis of the TB Fast Track Trial
Source: Open Forum Infect Dis. 2022 Jun 9;9(7):ofac265. doi: 10.1093/ofid/ofac265 (PMC9290545; doi:10.1093/ofid/ofac265)
Supplement: ofac265_Supplementary_Data [file ofac265_supplementary_data.zip › tbft_rf_suppmaterial_20220504_v5.docx]

**Supplementary material**

**Risk factors for hospitalisation or death among adults with advanced HIV at enrolment for care in South Africa: a secondary analysis of the TB Fast Track trial**

1. **Supplementary methods**

**Participants were deemed too unwell to be managed on an ambulatory pathway if any one of the following criteria were met:**

- Temperature >39˚C
- Respiratory rate >30 breaths/minute
- Heart rate >120 beats/minute
- Systolic blood pressure >180 mmHg or <90 mmHg
- Any other medical condition necessitating immediate hospital referral

**Clinical definitions for BMI and anaemia are shown in Supplementary table 1.1 and 1.2.**

**Principal components analysis to generate socio-economic position score**

A number of measures of SEP were available including possession of a number of durable assets, economic measures, housing characteristics, utilities and education. Input variables are shown in supplementary table 1.3. Briefly, descriptive analyses suggested all variables were relevant; three were dropped due to clumping. Internal coherence of the first component as a measure of SEP was appraised before a score was created and grouped into quintiles.68

PCA was used to construct a socio-economic position (SEP) score to incorporate a range of determinants of SEP, using the individual variables contained within the dataset. This was selected over simple sum of assets as it was felt that asset ownership may not fully reflect SEP uniformly across the study population due to different weighting of assets between population groups, for example livestock ownership in rural areas or ownership of few large/permanent assets in more mobile, urban populations.^68^

Descriptive analyses of all variables were conducted to identify clumping and truncation. Few individuals owned a donkey/horse, motorcycle/scooter or landline telephone (each <2%). The first two were not included in PCA, the third was incorporated into a binary variable for ‘phone of any type’ (landline and/or mobile telephone). Small groups were added to a similar category if one was apparent (e.g. informal shack and informal squatter) and ‘other’ free-text responses grouped into a relevant category where possible. Employment status and main source of income were collinear among those in full-time employment, however considerable variation in source of income amongst those in other employment categories was seen. It was inferred that this represented income from another member of the household, but as both variables were deemed to be relevant to SEP, both were retained. There were few missing data, only observations with data on all SEP variables were included in the PCA.

PCA was run using the correlation matrix and first principal component extracted as a measure of SEP (explaining 13% variation) and assessed for internal coherence. The distribution of scores followed a normal distribution and was grouped into quintiles.

**Stata code for multivariable models**

** BASIC MODEL **

stcox i.gendern i.agecat3 ib2.cd4catn i.district if lamcat!=. & bmiX!=. & ntbsx!=. & tbanytest!=., base

** BMI MODEL **

stcox ib2.bmiX i.gendern i.agecat3 ib2.cd4catn i.district if lamcat!=. & ntbsx!=., base

** TB MODEL **

stcox i.lamcat i.gendern i.agecat3 ib2.cd4catn i.district if bmiX!=. & ntbsx!=., base

stcox i.ntbsx i.gendern i.agecat3 ib2.cd4catn i.district if lamcat!=. & bmiX!=., base

** RISK FACTOR MODEL **

stcox ib2.bmiX i.ntbsx i.lamcat i.gendern i.agecat3 ib2.cd4catn i.district, base

** HB MODEL **

stcox ib3.hbgen i.agecat3 i.gendern c.cd4catn i.district c.bmiX i.anysx c.ntbsx i.lambin c.lamcat, base

1. **Supplementary results**

**Supplementary table 2.1:** Minimally adjusted hazard ratios for hospitalisation/death (n=1456)

**Supplementary figure 2.1:** Kaplan-Meier curves illustrating hospitalisation/death events over 6 months of follow up, stratified by key risk factors for poor outcomes.

1. **References**

1. World Health Organization. Haemoglobin concentrations for the diagnosis of anaemia and assessment of severity. Geneva, Switzerland: World Health Organization (WHO), 2011.

2. World Health Organization. Obesity: preventing and managing the global epidemic (Report of a WHO consultation). Geneva, Switzerland: World Health Organization (WHO), 2000.
