# Supplementary material for: Risk Factors for Hospitalization or Death Among Adults With Advanced HIV at Enrollment for Care in South Africa: A Secondary Analysis of the TB Fast Track Trial
Source: Open Forum Infect Dis. 2022 Jun 9;9(7):ofac265. doi: 10.1093/ofid/ofac265 (PMC9290545; doi:10.1093/ofid/ofac265)
Supplement: ofac265_Supplementary_Data [file ofac265_supplementary_data.zip › tbft_rf_supptables_20220504_v5.docx]

**Supplementary material**

**Risk factors for hospitalisation or death among adults with advanced HIV at enrolment for care in South Africa: a secondary analysis of the TB Fast Track trial**

Supplementary table 1.1: WHO classification of body mass index (BMI) for adults (≥19 years)^1^

| **BMI,** kg/m^2^ | **Interpretation** |
| --- | --- |
| <17 | Moderate-severe underweight * |
| 17–18.4 | Mild underweight |
| 18.5–24.9 | Normal range |
| 25–29.9 | Overweight |
| 30+ | Obese |
| * Severe underweight=BMI <16 kg/m^2^. Categorised together for this analysis due to sparse data in the lowest category BMI: body mass index; WHO: World Health Organization | |

Supplementary table 1.2: WHO classification of anaemia for adults^2^

| **Haemoglobin,** g/dL | | **Interpretation** |
| --- | --- | --- |
| **Men** | **Women *** |  |
| <80 | <80 | Severe anaemia |
| 80–109 | 80–109 | Moderate anaemia |
| 110–129 | 110–119 | Mild anaemia |
| ≥130 | ≥120 | Non-anaemia |
| * Refers to non-pregnant women. The classification for pregnant women is different and not shown as not of relevance to this study. WHO: World Health Organization | | |

Supplementary table 1.3: Variables used in principal components analysis to create socio-economic position index

| **Variable** | **Categorisation** |
| --- | --- |
| **Economic characteristics** | |
| Main income source | Formal employment, Self-employment, Odd jobs, Government grants, Income from investments, Student, Pensioner, No income, Other (17)* |
| Average household income | <R600, R601-1000, R1001-2000, R2001-4000, >R4000, not known** |
| Employment status | Employed full time, Self-employed, Employed part time, Student, Pensioner, Unemployed, Informal employment, Other (13)* |
| **Durable assets** | |
| Assets  (18 variables) | Binary variables for ownership of each of: Stove, Vacuum cleaner, Washing machine, Satellite TV, DVD player, Motor car, Mail postbox/bag, Mail delivery at home, Radio, TV, Computer, Refrigerator, ~~Landline telephone~~, ~~Mobile telephone~~, Bicycle, ~~Motorcycle or scooter~~, ~~Donkey or horse~~, Livestock. Variable for total number of assets also included. |
| **Housing characteristics** | |
| Dwelling type | House on stand, Traditional dwelling, Flat/Apartment, Cluster house, Townhouse, Semi-detached house, House/flat/room in backyard, Informal shack, Informal squatter, Room/Flat on property, Homeless, Other (2) |
| Main floor material | Natural floor, rudimentary floor, Finished floor |
| Main wall material | Plastic or cardboard, Mud, Mud and cement, Corrugated iron, Prefab or wood, Bare brick, Plaster or finished, Other (7)* |
| Occupants’ status in house | Owned/paid off, Owned/not paid off, Rented, Rent-free |
| **Utilities and infrastructure** | |
| Toilet facilities | Flush toilet, Septic tank, Chemical toilet, Ventilated Pit Latrine, Pit toilet without ventilation, Bucket, None |
| Main source of drinking water | Piped water inside, Piped water in yard, Piped water on stand, Borehole water, Open source, Other |
| **Education** | |
| Highest educational level attained | None/Pre-school, Grade 1-3, 4-7, 8-11, 12, Matric/Diploma^A^, BA, Other* |
| * Review of free-text responses enabled ‘other’ to be amalgamated into relevant categories prior to PCA.  ** Retained as a category in PCA  Strike-through indicates variables that were not included in the PCA (landline telephone and mobile telephone were combined, others were dropped). Other variables were grouped as described in text  ^A^ Matric=South African school-leavers’ exam normally taken at end of grade 12. | |

**Supplementary table 2.1:** Minimally adjusted hazard ratios for hospitalisation/death (n=1456)

|  | **Minimally adjusted model** | | | **Clinical model** | | |
| --- | --- | --- | --- | --- | --- | --- |
|  | **aHR** | **95% CI** | **p** | **aHR** | **95% CI** | **p** |
| **BMI /** kg/m^2^ | | | |  |  |  |
| <17 | 2.98 | (1.85­–4.79) | <0.001 ^a^ | 2.66 | (1.64–4.30) | 0.001 ^b^ |
| 17–18.4 | 1.79 | (1.03–3.13) |  | 1.61 | (0.92–2.81) |  |
| 18.5–24.9 | 1.43 | (0.98–2.09) |  | 1.38 | (0.95–2.02) |  |
| ≥25 | *ref* |  |  | *ref* |  |  |
| **TB-related symptoms** | | | |  |  |  |
| 0 | *ref* |  | <0.001 ^a^ | *ref* |  | <0.001 ^b^ |
| 1 | 1.34 | (0.91–1.97) |  | 1.33 | (0.90–1.95) |  |
| 2 | 2.48 | (1.69–3.64) |  | 2.4 | (1.63–3.53) |  |
| ≥3 | 2.35 | (1.51–3.63) |  | 2.07 | (1.33–3.23) |  |
| **Abbreviations:** aHR=adjusted hazard ratio; CI=confidence interval; p=p-value from likelihood ratio test (LRT) for association; BMI=body mass index; TB=tuberculosis.  **‘Minimally adjusted model’** is adjusted for sex, age and cluster (fixed effect for district) and restricted to observations included in model B (see main text and table 2).  **‘Clinical model’** is adjusted for sex, age and cluster (fixed effect for district) and variables shown. For male (vs female) aHR 0.95 (95% CI 0.72–1.27). For age 30–44 years and ≥45 years (vs 18–29 years), aHR 1.49 (95% CI 1.00–2.24) and 1.33 (95% CI 0.83–2.16) respectively.  ^a^ p<0.001 for linear trend and p>0.1 for departure from linearity from LRT.  ^b^ p<0.001 for linear trend and p>0.1 for departure from linearity from LRT; apart from TB-related symptoms where p=0.08 for departure from linearity. | | | | | | |
